# Estimating the number of homeless deaths in France, 2008–2010

**DOI:** 10.1186/1471-2458-14-690

**Published:** 2014-07-07

**Authors:** Cécile Vuillermoz, Albertine Aouba, Lise Grout, Stéphanie Vandentorren, Fanny Tassin, Layla Vazifeh, Walid Ghosn, Eric Jougla, Grégoire Rey

**Affiliations:** 1 Inserm, CépiDc, Le Kremlin-Bicêtre, France; 2 Inserm, UMRS 1136, Pierre Louis Institute of Epidemiology and Public Health, Department of Social Epidemiology, Paris, France; 3 Department of Social Epidemiology, Pierre Louis Institute of Epidemiology and Public Health, Sorbonne Universités, UPMC Univ Paris 06, UMRS 1136 Paris, France; 4 Le Collectif Les Morts de la Rue, Paris, France; 5 Observatoire du Samu Social, Paris, France; 6 Observatoire National de la Pauvreté et de l’Exclusion Sociale, Paris, France

**Keywords:** Homeless, Mortality, Capture-recapture, Completeness

## Abstract

**Background:**

The homeless population of France has increased by 50% over the last 10 years. Studies have shown that homelessness is associated with a high risk of premature death. The aim of this study was to estimate the number of homeless deaths in France between 2008 and 2010, using a reproducible method.

**Methods:**

We used the capture-recapture method to estimate the number of homeless deaths in France using two independent sources. An associative register of homeless deaths was matched with the national exhaustive database of the medical causes of death, using several matching approaches based on various combinations of the following variables: gender, age, place of death, date of death.

**Results:**

The estimated number of homeless deaths between 2008 and 2010 was 6730 (95% CI: [4381–9079]), a number greatly underestimated by the two sources considered separately (less than 20%).

**Conclusions:**

In the absence of a register of the homeless deaths, the capture-recapture method provides an order of magnitude for evaluation of the resources that may be allocated by policy makers to manage the issue. Based on common and routinely produced databases, this estimate may therefore be used to monitor the mortality of the homeless population. Further studies about homeless mortality, particularly on the lead causes of deaths, are needed to manage this issue and to implement strategy to decrease the number of homeless deaths.

## Background

Homelessness is a growing problem in France. The French National Institute for Statistics and Economic Studies (INSEE) reported a 50% increase in the homeless population over the last decade and estimated there were 144,000 homeless people in 2012 [1]. Homelessness is defined as having, on a given day, used an accommodation service or slept in a place not meant for human habitation (street, makeshift shelter, etc.) [2]. Given the potentially heterogeneous features of the homeless population, policy makers need indicators to evaluate the consequences of such rapid growth on health, in order to legitimately allocate the required resources to manage the issue. Among the health indicators, mortality is a strong component of evaluation.

Homelessness constitutes a high mortality risk factor. Studies of homeless mortality, mainly in the United States [3-10], Canada [11-14], Europe [15-21] and Australia [22] conducted since 80’s, found that mortality rates among homeless were 3 to 13 times higher than rates in the general population. The causes of deaths are different from one study to another but the leading causes are cardiovascular diseases, accident, intoxication, and suicides. However, the number of homeless deaths occurring each year in France had still not been documented [23].

In the absence of a register of the homeless in France, a capture-recapture approach was used to estimate the number of homeless deaths. The method is considered to be the most suitable method of generating reliable estimates of hard-to-reach populations for which at least two data sources are available [24,25].

An exploratory study [23] identified two sources of mortality data on the homeless population at national level in France: the CépiDc (The French Epidemiological Center for the Medical Causes of Death, which reports to the French National Institute for Health and Medical Research) and *'Le Collectif les Morts de la rue*' ('deaths in the street' association, subsequently referred to as the CMDR) databases. CépiDc collects all the death certificates for deaths in France and is responsible for the analysis of the causes of death. Some of the certificates indicate 'homelessness' but this status is not systematically reported. The CMDR specifically collects data on the deaths of homeless people in order to alert politicians on their health conditions. It is considered to be the most exhaustive source of homeless death records, although the completeness of the data is not known.

The aim of this study was to estimate the number of homeless deaths in France between 2008 and 2010 using a reproducible method applied to the two data sources.

## Methods

### The CMDR database

The CMDR collects data on homeless deaths in order to implement the means and actions needed for research and the reporting of violent causes of homeless deaths (street homeless and those living in homeless shelters), to ensure dignified burial, and accompany the people in mourning. The information collected is mainly based on various informal reporting circuits: associations addressing to homeless, institutions, relatives and media. Associations, institutions or relatives could report information about the death by filling a form, available on the CMDR website, or by informal means.

In cases where the CMDR learn the death by media or when data are missing, the CMDR contacts services addressing to homeless people in order to learn more about the death. From January 2008 to December 2010, 1145 homeless deaths were registered in this database (source A).

### The CepiDc database

The death certificate is composed of two parts: administrative and medical parts. INSEE manages the administrative part that contains the name of the dead person and information relating to civil status. CépiDc manages the non-nominative medical part which contains the medical causes of death, dates of birth and death, and cities of residence and death. From January 2008 to December 2010, CépiDc registered and coded a total of 1.6 million deaths for the entire population of France. For 241 of the deaths, 'homelessness' (street homeless and those living in homeless shelters) was reported (source B). This type of information is coded using the International Classification of Diseases (10th revision - ICD-10) as 'Z59.0', in the section 'Factors influencing health status and contact with health services'. According to the World Health Organization (WHO) guidelines, medical certifiers are to report homelessness if they consider that it contributed to a person’s death.

Information available in both databases included age, sex, birth and death dates, city of death, birth and death places (housing, hospital, street…), and causes of death.

Access to the two databases is not freely available. Overall, for each database, a specific permission is needed. This study has been approved by the French Commission for Data Protection and Liberties (Commission Nationale de l'Informatique et des Libertés: CNIL).

### The capture-recapture method

When two sources are considered, the total number of deaths, N, is estimated with numbers of deaths in each source NA and NB and number in common NAB [26] as:

$$N=\frac{N_{A}\times N_{B}}{N_{\mathrm{AB}}}$$

Applying the capture-recapture method requires some general validity conditions:

– *Independence of sources* (the probability that an observation is in one of the two sources does not depend on the probability of it being in the other source): Since different actors produce the sources, the independence is plausible by construction. In the absence of more than two sources, the qualitative assessment of the dependency between the sources could not be implemented.

– *Adequate matching* (deaths designated by a source can be matched to those reported by another source without mismatched data): Given the strict rules of anonymity of the CépiDc database, this assumption can hardly be assessed.

– *Capture homogeneity* (all persons in the population have the same chance of being observed in any source): The homogeneity of the capture was studied by comparing the distributions of homeless deaths from source A and source B, by gender, age, season, place and region of death. When the distribution of the deaths for those variables was significantly different, stratified estimation was undertaken [24].

– *Closed population (*no movement of subjects within the population): since the study population consisted of dead people, this assumption was fulfilled.

### Identification of common cases among sources

Using a capture-recapture method requires matching the records from two sources in order to identify those in common. In the absence of any direct identifier in the CépiDc database, matching could only be performed indirectly with a set of variables common to both databases: age, gender, place of death, date of death.

However, the CMDR database has many missing or inaccurate records that prevent an optimal matching process. As the entire CépiDc database is exhaustive, a preliminary matching of source A and the entire CépiDc database (1.6 million records) was performed. This matching was composed of several steps. As the day of death and age constituted necessary information to avoid duplicates, the matching sample was divided into 4 groups according to the presence or absence of these two variables. For each sample, variables were included into the combination if the completeness rate was higher than 80%. For each death reported in the CMDR database, the algorithm searched for a correspondence in the CépiDc database. If no observation with the same combination into the two files was found, the algorithm allowed partial matches: one of the variables was sequentially removed and a new matching searched one or more observations with new combinations. In the case where several observations of CépiDc were consistent with a single death of the CMDR database, only the most informative combination of matched deaths was kept.

Out of the 1145 deaths of the source A, 391 were not retrieved from the CépiDc database due to missing or inaccurate data and were excluded from subsequent calculations. Thus, the capture recapture method was applied to the source A’(N = 754), excluding the 391 deaths of the 1145 of the source A. Finally, the total number of deaths, N, was estimated from the numbers of deaths in each source N_A'_ and N_B_ and from the number of deaths simultaneously present in both sources N_A'B_[26]. Here, A' is the set of the 754 deaths from the CMDR database (matched to the entire CépiDc database), B is the set of the 241 homeless deaths from the CépiDc database, and A'B is the set of deaths present in both sources.

The data completeness for each source was calculated as the ratio of the total number of homeless deaths in each source and the total capture-recapture estimated deaths.

## Results

**Figure 1:**
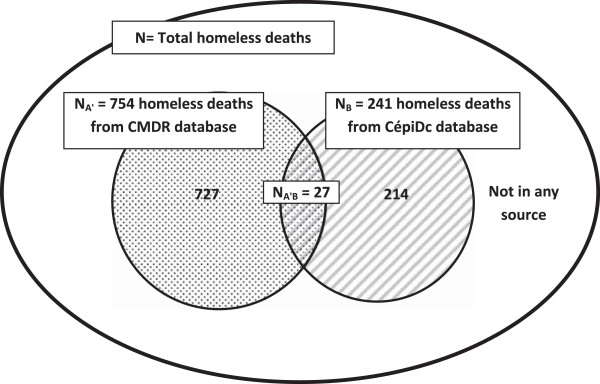
The capture-recapture method applied to the CMDR and CépiDc databases.

The distributions of homeless deaths differed significantly between source A' and source B (p < 0.0001) by region only (data not shown). The main differences were observed for the Paris region and for the North where the proportions of homeless deaths were 48% and 9%, respectively, in source A' versus 24% and 20% in source B.The overall number of common cases was then estimated to be 27 deaths (Figure 1).

According to the capture-recapture method, the total estimated number of homeless deaths in France was 6730 (95% CI [4381-9079]) over the period 2008–2010 (Table 1).

**Table 1:** Estimating the total number of homeless deaths and data completeness of the main sources recording homeless deaths

	**Observed deaths**			**Estimates**	**Data completeness of each source**		
	**N**_**A'**_	**N**_**B**_	**N'**_**AB**_	**N [95% CI]**	**Source A'**	**Source B**	**CMDR (entire)**
Paris area	360	57	10	2052 [913–3191]	18% [11–39]	3% [2–6]	24% [16–54]
Outside Paris area	394	184	17	4264 [2375–6154]	9% [6–17]	4% [3–8]	15% [10–27]
Total of strata*	754	241	27	6316 [3967–8665]	12% [9–19]	4% [3–6]	18% [13–29]
Global	754	241	27	6730 [4381–9079]	11% [8–17]	4% [3–6]	17% [13–26]

*Sum of estimated numbers by region.

When the estimate was stratified by region, the total estimated number of deaths was very similar to the overall estimate (6316 deaths 95% CI [3967–8665]).

Overall, the data completeness of the homeless deaths in the CMDR database was 11%. Only 4% of homeless deaths were reported as such on the death certificates. For the entire CMDR database, considering the total of 1145 deaths and not only the 754 deaths, the data completeness was 17%. In the CMDR database, the data completeness was two-fold greater in the Paris region than elsewhere.

## Discussion

The capture-recapture method using two sources enabled estimation that about 6000 homeless people died in France from January 2008 to December 2010, i.e. around 2000 deaths per year, with a large confidence interval. The number of homeless deaths was greatly underestimated by the two sources, CMDR and the CépiDc, taken separately. The CépiDc could not probably enhance the completeness of the collection of the homeless deaths since the only solution would be to introduce a specific location for the homeless status on the death certificate. On the contrary, in view of the missing data in the CMDR database, the CMDR has planned to develop its network to increase the completeness and standardization of its database. The estimation could be more accurate by enhancing the matching rates and therefore by improving the quality of the CMDR data. The age and the day of death constituted necessary information to avoid duplicates. The CMDR has planned to implement strategies to improve the quality of these data, particularly regarding age and date of death. One solution to improve the matching would be to provide a unique identifier but the medical section of the death certificate is anonymous and confidential.

The only recent estimate of the homeless population of France was made by the INSEE, which reported that there were 144,000 homeless people in 2012 [1]. In 2001, the first national survey of the homeless had estimated there were 86,500 homeless people in France. In the period of our study (2008–2010), the size of the homeless population was unknown. The structures and size of homeless population are constantly evolving. In particular, in recent years, homeless families become the fastest growing segment of the homeless population in Paris area [27]. Thus, estimates are only valid for the dates they are conceived for. In addition, the definition of the homeless population in the present study was not the same as for the INSEE study: it did not include people sleeping rough and not using institutional facilities, or homeless people living in towns with a population of less than 20,000. For this reason, the mortality rate of the homeless population could not be estimated in this study or compared with the international literature.

Considering the small number of deaths common to both sources, the estimates of the number of deaths are imprecise and should be considered with caution. However, the low data completeness of each source is plausible, first because the information network of the CMDR is highly dependent on local initiatives and does not cover the whole of France homogeneously, and secondly because medical death certifiers rarely consider homelessness to be relevant information to be reported with the causes of death.

Some of the general conditions that validate the use of a capture-recapture method were not completely fulfilled by this study. First, the definition of homeless people was not the same for the two sources. While the homeless deaths reported in CMDR included all forms of homelessness (defined by INSEE), the homeless deaths reported in the CépiDc database are those in which the physician considered that homelessness played a role in the death. The CépiDc homeless death definition is likely to be a subcategory of the overall definition of homeless death. However, the proportion of deaths with ICD code 'Z59.0' (homelessness) that was not retrieved in the CMDR database was unexpectedly large (89%). In addition, we supposed that homelessness plays a role in the majority of cases even if it is not the underlying cause of death. Homelessness is associated with high increased risks of all-cause mortality [15]. As such, in most of the cases the medical certifier should declare homelessness on the death certificate. However the low occurrence of homeless reporting on death certificate is likely to be attributable to the focus that medical certifier may put on medical rather than social conditions. Therefore, the definitions are not necessarily so different, and being caught by each of the two sources may be seen as independent random events.

This study showed some heterogeneity in the capture, specifically regarding the areas in which deaths occurred. For this reason, the estimates were stratified by the geographic area, but the results remained unchanged. The dependence between the two sources was not quantitatively evaluated but is likely to be low. However, a positive dependence would imply that the total number of homeless deaths was underestimated. The condition requiring perfect matching could not be met in this study. The matching algorithm was implemented in order to minimize the number of false matches by reducing the number of possible multiple matches between the CMDR and the entire database. Excluding the 391 unmatched records from the calculation is equivalent to assuming that the proportion of death certificates with the 'homeless' code in the CépiDc record was the same as that for the matched records, which seems the most reasonable option.

This study represented the first part of a broader project whose objective was to describe the mortality among the homeless population in France. The next step is to describe the deaths characteristics (age, location, season and causes of deaths) and to compare to the mortality in French population, in order to implement efficient strategy, at national level, provided by policymakers and public health professionals serving this population, and not only through local initiatives.

## Conclusion

Knowledge of the number of homeless deaths in a population is necessary to monitor this public health issue. This study proposed a possible way to estimate the number of homeless deaths by using a capture-recapture method which should overcome the current limitations. As a national cause of death register and associations aiding homeless populations exist in most industrialized countries, similar sources may be accessible to public health actors. Further studies about homeless mortality, particularly on the lead causes of deaths, are needed to manage this issue and to implement strategy to decrease the number of homeless deaths.

## Competing interests

The authors declare that they have no competing interests.

## Authors’ contributions

CV and GR were the principal investigators of the study. CV was in charge of the calculation, contributed to the interpretation of the data and drafted the manuscript. GR provided epidemiological expertise and participated in the interpretation of the data. AA, EJ, LG, SV and WG participated in the interpretation of the data. LV participated in the causes of death data expertise. All the authors read and approved the manuscript.

## Pre-publication history

The pre-publication history for this paper can be accessed here:

http://www.biomedcentral.com/1471-2458/14/690/prepub
